# Successful surgical resection of a complicated left ventricular diverticulum in a neonate presented with unexplained anemia: a case report

**DOI:** 10.1097/MS9.0000000000000212

**Published:** 2023-03-24

**Authors:** Oadi N. Shrateh, Muttaz M.M. Abusamra, Majdi A. Abudaoud, Moayad A. Srour, Nizar Hijjeh, Nidal Haymouni, Iyad Sbeitan, Samer Abdelraziq, Mohammed Abutaqa

**Affiliations:** a Al-Quds University School of Medicine; b Department of Pediatric Cardiology, Al-Makassed Islamic Charitable Hospital, Jerusalem, Palestine

**Keywords:** case report, congenital heart defects, diverticulum rupture, left ventricular diverticulum, surgical resection, unexplained anemia

## Abstract

**Introduction:**

Congenital left ventricular diverticulum (LVD) is a rare congenital cardiac anomaly and may be complicated by fatal adverse events such as diverticulum rupture. Most LVD cases are asymptomatic and often discovered incidentally. Herein, we describe an unusual and peculiar clinical presentation with felicitous surgical management of ruptured LVD.

**Case presentations:**

A 10-day-old male infant presented with severe, intractable, and unexplained anemia associated with respiratory distress. Upon admission, the patient was clinically shocked with a hemoglobin level of 6.0 g/dl. As chest imaging showed cardiomegaly, echocardiography was performed and revealed a 9×10 mm diverticulum arising from the posterolateral wall of the left ventricle along with blood and clot collection in the pericardium. The patient underwent an urgent surgical resection of the diverticulum. He was followed up for 2 years without any readmissions or cardiac complaints.

**Clinical discussion:**

Systemic thromboembolism, heart failure, infarction, and tachyarrhythmias have all been reported as complications of LVD. The most serious complication is diverticulum rupture, which can result in death. As a result, this congenital defect should be discovered early to determine the potential risks and plan appropriate treatment.

**Conclusion:**

Congenital heart defects such as LVD should be suspected in neonates presenting with unexplained and intractable anemia. To avoid the diagnosis confusion and risk of serious complications in LVD patients, such as spontaneous rupture of the diverticulum, we advocate immediate surgical management of LVD in children.

HIGHLIGHTS

- Congenital left ventricular diverticulum (LVD) is a rare congenital cardiac anomaly.
- Severe, intractable, and unexplained anemia in neonates may indicate a serious underlying congenital heart anomalies such as LVD.
- Most cases are asymptomatic but may be complicated by fatal adverse events.
- The most serious complication is diverticulum rupture, which can result in death.
- We advocate that surgical resection of all LVD is a more convenient and safe option compared with conservative management.

## Introduction

Congenital LVD is a rare congenital anomaly with an overall incidence of 0.4%, or three in 750 autopsies of congenital heart defects^1^. The diverticulum of the left ventricle is defined as a swollen structure that contains the endocardium, myocardium, and pericardium and exhibits normal systolic contractility^2^,^3^. Since it is commonly associated with other cardiac and midline thoracoabdominal malformations, LVD is frequently diagnosed in early infancy^4^. The vast majority of LVD cases are clinically silent and are often encountered incidentally on physical assessment for other reasons. Although these anomalies are usually benign, rare fatal complications such as thromboembolism, cardiac arrhythmia, heart failure, and diverticulum rupture have been reported^5^. Therefore, prompt recognition and early surgical intervention are critical, as delayed diagnosis and treatment of such complications may be associated with poor prognostic outcomes. Herein, we report a case of a 10-day-old infant who presented with severe, intractable, and unexplained anemia who was found to have a ruptured congenital LVD. The patient underwent a felicitous and successful surgical resection of the diverticulum with an uneventful 2-year postoperative follow-up period. This case report has been reported in line with the SCARE Criteria^6^.

## Case presentation

**Figure 1:**
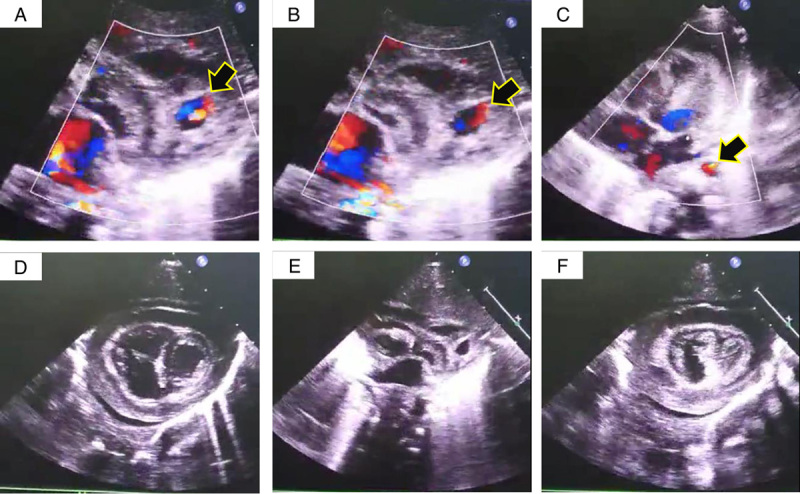
Echocardiography showing the left ventricular diverticulum (arrows in A–C), and hemopericardium with blood clots (in D–F).

**Figure 2:**
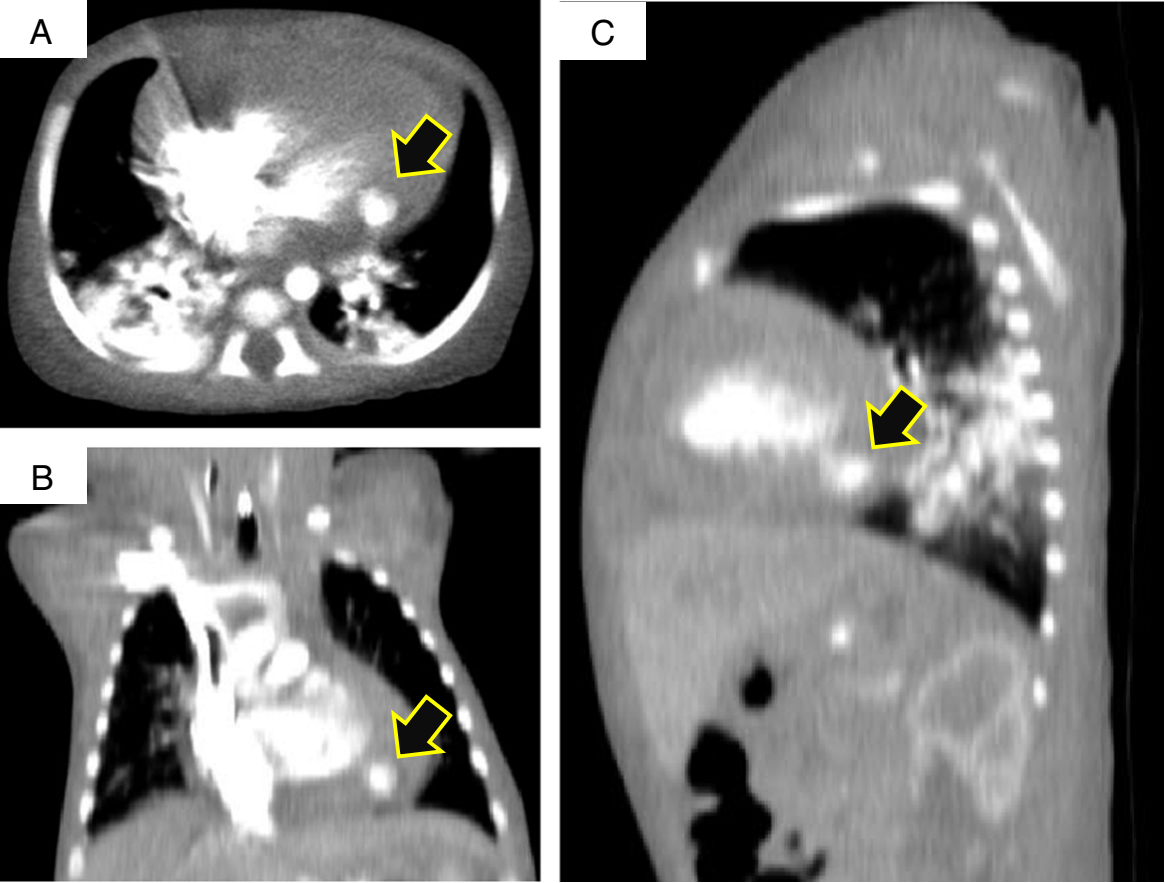
Enhanced computed tomography scan showing a contrast-filled left ventricular outpouching (arrows); with in an axial view (A), in a coronal view (B), and in an oblique view (C).

A 10-day-old male infant was referred to our hospital for the assessment of severe, intractable, and unexplained anemia associated with respiratory distress. The patient’s parent reported that their child has no personal and/or family history of cancer, any acute, repeat, or discontinued medications, any allergies, any genetic or psychosocial issues, and has a free past surgical history. Upon admission to the hospital, the patient was in apparent respiratory distress and clinically shocked with a hemoglobin level of 6.0 g/dl, which was treated by packed red blood cells administration in the transferring hospital without significant improvement. Physical assessment was normal except for tachycardia without heard murmurs and hypotension. When a plain chest roentgenogram was performed and demonstrated cardiomegaly, a transthoracic echocardiogram was planned. The echocardiography carried out in our hospital revealed the presence of pericardial effusion of 5–8 mm in diameter with scattered blood clots and a diverticulum with dimensions of nearly 9×10 mm arising from the posterolateral wall of the left ventricle near the base of the heart (Fig. 1). We suspected that microperforation of the diverticulum was the reasonable source of the blood and clots in the pericardium. A color Doppler study demonstrated pulsatile flow in the diverticulum. An enhanced computed tomography scan performed before surgery revealed a 9 mm contrast-filled left ventricular outpouching (Fig. 2). The patient was diagnosed with congenital LVD complicated by rupture. The imaging diagnosis was confirmed further during intraoperative exploration. The patient was arranged for emergent surgical resection of the ruptured diverticulum. The procedure was performed by a consultant at the department of pediatric cardiac surgery and congenital heart defects at a tertiary referral hospital. The patient was followed up for 2 years without any readmissions or cardiac complaints and the parents adhered to and tolerated the provided advices.

**Figure 3:**
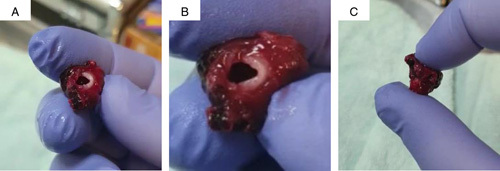
The resected left ventricular diverticulum.

## Operative and postoperative course

The patient underwent single-lumen intubation. An arterial line, right internal jugular central venous line, nasogastric tube, and Folly’s catheter were inserted. Midline sternotomy and thymectomy were performed. Upon opening of the pericardium, huge clots surrounding the heart were observed. Heparin was infused; cannulation of the aorta and right atrium; institution of cardiopulmonary bypass; aortic clamp; and anterograde custodial cardioplegia were done. After that, the hemopericardial collection was evacuated. Elevation of the inferoposterior surface of the heart disclosed the LVD. Ligation and excision of the diverticulum (Fig. 3) were carried out. Histopathologic assessment of the excised specimen confirmed the diagnosis. Rewarming, smooth heart rebate, deairing off cardiopulmonary bypass, decannulation, and insertion of one mediastinal drain were performed. The sternum and skin were closed with vicryl and monocryl, respectively.

The patient was transferred intubated to the pediatric cardiac intensive care unit. The patient was hemodynamically stable with no need for pressor support. A cardiac echocardiogram on the postoperative day 1 showed normal global function and geometry with no residual outpouching, pericardial effusion, or vegetation. A postoperative cardiac computed tomography scan revealed that the LVD had completely disappeared and that the morphology was normal. The patient was extubated on postoperative day 2, breastfeeding was restarted on the same day, and the mediastinal chest tube was removed on postoperative day 3 with significant improvement in respiratory distress. The patient was re-examined at 1, 3, 6 months, 1 year, 18 months, and 2 years in a 2-year postoperative period during which an ECG showed no arrhythmias. There were no readmissions to the hospital due to cardiac complaints, and no other adverse events occurred.

## Discussion

The LVD is a rare congenital left ventricle malformation. It is described as a bulging structure in the left ventricle containing all three layers of the cardiac wall, including the endocardium, myocardium, and pericardium, and exhibits synergistic function with the left ventricular contraction. This diverticulum is connected to the ventricular wall by a narrow neck ∼1 cm^2^,^7^. A meta-analysis study found 453 cases of LVD between the first description in 1816 and January 2012. There have only been a few reported cases of LVD since O’Bryan^8^ first identified it as a rare congenital heart defect in 1838, and the majority of them are in neonates and children^9^. The estimated incidence of LVD is 0.4%, or three in 750 autopsies of congenital heart defects^2^. Although the exact etiopathogenesis of the LVD is unclear, it is currently thought to occur as a consequence of impaired endocardial tube development during the fourth week of embryologic growth^10^. Some authors categorize LVD as either muscular or fibrous. Others classified it into two types based on the diverticulum’s anatomical location: apical, which is usually associated with other congenital cardiac anomalies, and nonapical, which is mostly isolated. The LVD is also divided into congenital and acquired^3^,^5^,^11^. Our patient had a congenital, fibrous, and nonapical LVD.

Because it is commonly associated with other heart and thoracoabdominal abnormalities, LVD is most often detected in early childhood^12^,^13^. Cantrell syndrome is defined as a constellation of congenital malformations including LVD, congenital cardiac defects, midline congenital anomalies, substernal abnormalities, diaphragmatic defects, and incomplete formation of pericardium^14^. However, isolated LVD may occur in up to 30% of cases^15^. Although they are exceptionally rare, it is important to distinguish LVD from other left ventricular outpouchings (bulge or pseudoaneurysm) in terms of location, structure beneath the ventricle’s three layers, lack of contraction, or paradoxical contraction in comparison with ventricle muscle^5^,^13^.

Given the rarity of congenital LVD, its natural history has not been thoroughly studied. The majority of cases are asymptomatic and were identified incidentally during a physical assessment for other reasons, a few of which can be discovered prenatally using echocardiography^15^,^16^. A four-chamber view of the heart can be used to make a prenatal diagnosis of such ventricular defect, especially if it is large. There are few reported clinical reports enumerating the defect, so information on the natural course of ventricular diverticulum identified during fetal life is limited^17^. In comparison to diverticuli, fetal ventricular aneurysms detected in early pregnancy may have a poor prognosis, according to the size and advancement of the lesion^18^. Two out of three fetuses with left ventricular aneurysm developed hydrops and died in utero in a study of seven fetuses with ventricular diverticula or aneurysms. Three of the four cases with diverticula had significant pericardial effusion. After 8–24 months of postnatal follow-up, one baby with aneurysm and all infants with diverticula remained symptomless^19^. The vast majority of diverticula have very few but may be fatal complications. According to a comprehensive study evaluating the clinical sequalae of LVD, 2.9% of 453 cases had cerebral or peripheral vascular complications, 9.9% had cardiac arrhythmias, 6.8% had complications of heart failure, and 4.2% of cases had rupture of the diverticulum, with the vast majority of ruptured cases (90%) occurring in children under the age of 18^15^.

Although there is no consensus on the optimal way to manage LVD, some scholars presume that asymptomatic LVD can be monitored without requiring immediate surgery. Notwithstanding, other scholars believe that surgical resection of all LVD is a more convenient and safe option compared with conservative management, considering the possibility of a left ventricular rupture and other devastating complications, including systemic thromboembolism, or compromised ventricular function, and tachyarrhythmias^10^,^20^–^22^. A case series included 12 neonates with LVD, who were managed conservatively, noticed that ten patients had at least one adverse consequence, including two cases of spontaneous diverticulum rupture^23^.

Here, we report a case of a patient with isolated congenital LVD who presented with an unusual and enigmatic clinical manifestation as a severe, intractable, and unexplained anemia. The patient also experienced shock with respiratory distress. After a thorough investigatory process, he was found to have a complicated LVD by rupture with a resultant hemopericardium that explained the underlaying mechanism of the patient’s anemia. The patient successfully underwent subsequent surgical resection of the LVD with an unremarkable postoperative period.

## Conclusion

LVD is a rare type of congenital cardiac outpouchings that’s often incidentally detected. Although it’s rarely symptomatic, LVD may present with an unusual and peculiar manifestation such as unexplained anemia, as in our case. Most cases of LVD have a few or no complications. However, some can result in irreversible and/or lethal consequences, including thromboembolism, arrhythmia, heart failure, or even rupture of the diverticulum. Accordingly, our experience, in this case, affirms the significance of elective surgical resection of all identified LVD, even if they are asymptomatic.

## Ethical approval

Our institution has exempted this study from ethical review.

## Consent

Written informed consent was obtained from the patient for publication of this case report and accompanying images. A copy of the written consent is available for review by the Editor-in-Chief of this journal on request.

## Sources of funding

None.

## Authors’ contribution

O.N.S.: writing the manuscript. O.N.S., M.M.M.A., M.A.A., M.A.S., N.H., I.S.: Imaging description. O.N.S., N.H., S.A., and M.A.: reviewing and editing the manuscript.

## Conflicts of interest disclosure

The authors declare that they have no financial conflict of interest with regard to the content of this report.

## Research registration unique identifying number (UIN)

None.

## Guarantor

Oadi N. Shrateh.

## Provenance and peer review

Not commissioned, externally peer-reviewed.
